# Physical and mental health of informal caregivers before and during the COVID-19 pandemic in the United States

**DOI:** 10.1186/s12889-023-17164-8

**Published:** 2023-11-27

**Authors:** Emery L Ngamasana, Meagan Zarwell, Laura H Gunn

**Affiliations:** 1 Department of Public Health Sciences, University of North Carolina at Charlotte, Charlotte, NC USA; 2 School of Data Science, University of North Carolina at Charlotte, Charlotte, NC USA; 3 Department of Primary Care and Public Health, Imperial College London, London, UK

**Keywords:** Informal caregiving, Physical health, Mental health, COVID-19

## Abstract

**Background:**

Informal caregiving, a common form of social support, can be a chronic stressor with health consequences for caregivers. It is unclear how varying restrictions during the COVID-19 pandemic affected caregivers’ physical and mental health. This study explores pre-post March 2020 differences in reported days of poor physical and mental health among informal caregivers.

**Methods:**

Data from the 2019/2020 Behavioral Risk Factor Surveillance System survey were used to match, via propensity scores, informal caregivers who provided care during COVID-19 restrictions to those who provided care before the pandemic. Negative binomial weighted regression models estimated incidence rate ratios (IRRs) and differences by demographics of reporting days of poor physical and mental health. A sensitivity analysis including multiple imputation was also performed.

**Results:**

The sample included 9,240 informal caregivers, of whom 861 provided care during the COVID-19 pandemic. The incidence rate for days of poor physical health was 26% lower (p = 0.001) for those who provided care during the COVID-19 pandemic, though the incidence rates for days of poor mental health were not statistically different between groups. Informal caregivers with low educational attainment experienced significantly higher IRRs for days of poor physical and mental health. Younger informal caregivers had a significantly lower IRR for days of poor physical health, but higher IRR for days of poor mental health.

**Conclusions:**

This study contends that the physical and mental health burden associated with informal caregiving in a period of great uncertainty may be heightened among certain populations. Policymakers should consider expanding access to resources through institutional mechanisms for informal caregivers, who may be likely to incur a higher physical and mental health burden during public health emergencies, especially those identified as higher risk.

**Supplementary Information:**

The online version contains supplementary material available at 10.1186/s12889-023-17164-8.

## Introduction

Robust scientific evidence links the functional dimensions of social support (i.e., perceived, received, emotional, belonging, tangible, and informational) to physical and mental health outcomes [1–3]. Informal caregiving, defined as care provided by unpaid persons to support a family member, other relative, or friends in old age or living with a chronic illness or disability, is a common form of social support [4–6].

The theory of caregiver stress derived from the Roy Adaptation Model [7] posits that an individual is an adaptive system affected by internal and external stimuli [8], which can be summarized in three categories: focal, contextual, and residual. Focal stimuli represent external factors that immediately confront the person (e.g., the caregiver’s objective burden). Contextual stimuli encompass identifiable factors (e.g., social support, stressful life events, social roles) that contribute to the effects of the focal stimulus. Finally, residual stimuli include factors that have unclear effects in the current situation (e.g., race, age, gender, or type of relationship). According to this theory, the duties or tasks associated with caregiving of a person with chronic illness (focal stimuli), activate a coping mechanism and prompt caregivers to seek available physical and psychological resources to cope with caregiving [8]. Consequently, caregivers may experience increased stress levels leading to adverse health outcomes [8–10].

Studies suggest that in normal circumstances informal caregiving may be a chronic stressor with deleterious consequences for caregivers’ physical and mental health. For example, caregiving can alter the immune system and trigger stress hormones [11] and increase the risk of depressive symptoms and higher perceived stress levels [5]. A meta-analysis compared the physical health of informal caregivers (ICs) with demographically similar non-caregivers and concluded that ICs exhibited a slightly greater risk for health problems (e.g., functional cellular immunity, stress hormones, antibodies, and global reported health) compared to non-informal caregivers, however, the clinical relevance of such differences remains elusive [12]. Although research has shown an all-cause mortality advantage of informal caregiving, this advantage is not evident when informal caregiving is operationalized precisely (i.e., provision of assistance with at least one activity of daily living or with an instrumental activity of daily living) versus broader measurements (e.g., provision of care to someone with a chronic physical or cognitive disability) [4]. Altogether, these findings suggest informal caregiving may be negatively impacting the physical and mental health of ICs. However, less is known about how caregiving stressors may be heightened during prolonged periods of societal stress such as during natural disasters or global pandemics [13, 14].

The COVID-19 pandemic disrupted the everyday functional mechanisms through which social support is exchanged worldwide [15]. A recent review of the literature on multigenerational relationships in the United States showed that COVID-19 simultaneously posed unique challenges to social support exchanged between generations within families and also may have provided opportunities for greater solidarity within families [16]. For example, a study included in the review found that in July 2020, about 52% of young adults (18–29 years old) resided with one or both of their parents, up from 47% in July 2019. The largest growth in young adults living with their parents was observed among younger (18–24 years old) and White adults [17]. The review also pointed that multigenerational family ties were impacted differently between sub-groups of the United States population, with Black and other ethnic minority groups being more likely to suffer severe health consequences compared to their White counterparts because of the social determinants of health. This disparity may have well impacted how social support is exchanged within families. A study by Millet et al. [18] found that as early as April 13th, 2020, counties with a predominant Black population reported at least one COVID-19 case (nearly 97%) and at least one death (49%), compared to 80% and 28% of all counties that were not predominantly Black. Thus, higher COVID-19 prevalence in these counties may have resulted in increased challenges for informal caregivers (e.g., excessive demands in daily tasks, tradeoff between informal caregiving and work for pay, etc.) [19]. Other studies reported increased informal caregiving responsibilities and poor mental health outcomes for women compared to men [20, 21]. Another study reported changes in caregiving tasks (e.g., new focus on vigilance and safety, keeping connected, etc.) [15].

Studies have also shown mental and physical health differences by sociodemographic characteristics of ICs. For instance, Brown and Cohen [22] found that although informal caregiving was positively associated with poor mental health irrespective of gender, male ICs exhibited a higher gradient of poor mental health compared to women. Another study by Do, Cohen, and Brown [23] found that income, race, and ethnicity significantly modified the relationship between informal caregiving and health. Evidence from these studies suggests that sociodemographic characteristics of ICs may be important confounders to account for in analyzing the association between informal caregiving and health.

In addition, characteristics of care recipients may influence the stressors experienced by ICs. Generally, elderly patients with terminal or chronic illnesses (e.g., dementia, autism spectrum disorders, Alzheimer’s, Parkinson’s disease, etc.) prefer home-based informal care because they cannot remain within the confines of the hospital for as long as their illness lasts [4, 24]. However, during the COVID-19 pandemic, guidance for contact with people living with a chronic or terminal illness in long-term care facilities changed frequently due to their higher risk classification [25]. For example, long-term care facilities implemented new and varying restrictions on visitations, use of personal protective equipment, vaccine requirements, and age restrictions for visitors due to concerns about transmission risk [26]. It is unclear how the evolving modifications to guidance, recommendations, and restrictions over the course of the COVID-19 pandemic have affected ICs’ physical and mental health.

The purpose of this study is to explore variations in the physical and mental health of ICs pre-COVID-19 through the pandemic period. To the best of our knowledge, no study has explored this question in a way that minimizes the risk of confounding variables that arise when two unbalanced groups are compared. Findings from this study carries significant policy implications, in terms of institutional mechanisms that could be enacted to support ICs who are more likely to incur a higher burden of poor physical and mental health during prolonged periods of societal stress. Consistent with the theory of caregiver stress, this study also investigates the extent to which demographic characteristics (e.g., race, age, biological sex, or type of relationship) modified the physical functioning and mental health of ICs.

## Methods

We used the publicly available cross-sectional data from the Behavioral Risk Factor Surveillance System (BRFSS), a nationally representative dataset of United States residents 18-years and older, that explore a series of questions about health-related risk behaviors, chronic health conditions, and use of preventive services [27]. Initiated in 1984 within 15 states, BRFSS now collects data on all 50 states as well as the District of Columbia and participating United States territories. In 2020, despite COVID-19-related disruptions, all 50 states, the District of Columbia, Guam, and Puerto Rico collected BRFSS data during each calendar month. Core measures assessed by the 2019 and 2020 BRFSS surveys included health status and healthy days. Both the 2019 and 2020 BRFSS surveys featured an optional module for ICs.

Despite potential disruptions in data collection for the 2020 survey, all states met the criterion for a probability sample, which allowed us to conduct a comparative study before (2019 through February 2020) and during the COVID-19 pandemic (March 2020 onwards) [27]. The 2019 and 2020 BRFSS surveys included 330,619 and 345,315 completed interviews, respectively.

In 53 states and territories, BRFSS teams contacted participants via random digit dialing and obtained verbal consent to participate in the study. Interviews were conducted using Computer-Assisted Telephone Interview (CATI). Further details can be found elsewhere [27].

### Predictor and covariates

**Relationship**. All respondents were asked “*During the past 30 days, did you provide regular care or assistance to a friend or family member who has a health problem or disability?*” Respondents who responded “*yes*” to informal caregiving were asked “*What is his or her relationship to you?*” Consistent with research on intergenerational family ties [16, 17, 28, 29], participant responses were organized into three categories: (1) sibling, spouse, or spouse siblings (husband, wife, brother in-law or brother, sister in-law or sister); (2) intergenerational (mother, father, child, grandmother, grandfather, grandchild, parent-in-law); and (3) other (another relative, friend). In the intergenerational family relationship literature, relationships between family generations are dyadic, representing two individuals from different family generations (e.g., relations between mothers and daughters or mother-in-law and sons-in-law) [28].

**COVID-19.** The exact survey date (month, day, year) and the length of time during which care was provided were used to create an indicator variable that determined whether the informal care started after March 13th, 2020, when the United States government proclaimed a National Emergency Declaration (NED), thereby triggering staggered nationwide lockdowns [30]. An indicator variable was coded to compare caregiving experiences before versus after the nationwide lockdown orders due to COVID-19. Those who provided care both before and after the COVID-19 NED were excluded from the analysis.

**Demographic characteristics.** The analyses controlled for race/ethnicity of the caregiver (i.e., Hispanic; non-Hispanic multiracial, non-Hispanic White, non-Hispanic Black, non-Hispanic who identified as other races), age group (i.e., 18–24, 25–34, 35–44, 45–54, 55–64, 65+), and biological sex assigned at birth (i.e., male or female). Analyses also controlled for level of educational attainment of the IC (i.e., did not graduate high school, graduated high school, attended college/technical school, graduated from College/Technical).

### Outcomes

The study defined two distinct outcomes by number of days, within the previous 30 days of: (1) poor physical health; and (2) poor mental health. Number of days of poor physical health were assessed by asking respondents “*Now thinking about your physical health, which includes physical illness and injury, for how many days during the past 30 days was your physical health not good?*” Likewise, number of days of poor mental health were assessed by asking respondents “*Now thinking about your mental health, which includes stress, depression, and problems with emotions, for how many days during the past 30 days was your mental health not good?*”

### Analysis

We performed a complete case analysis, including only respondents who were ICs in the previous 30-days, and who provided valid data on the abovementioned sociodemographic characteristics (i.e., age, biological sex, race/ethnicity, level of education, and relationship with the care recipient). A sensitivity analysis including hot-deck imputed data [31, 32] was also performed.

Propensity score matching was performed, using an optimal variable ratio matching of 1 treated to up to 2 controls, to simulate an experimental design with observational data, thereby creating exposed (provided informal care after NED) and control (provided informal care before NED) groups. Exact matches were performed on sex and approximate matches on age group; and additional covariates included race/ethnicity, education, and relationship with the care recipient. Standardized mean differences (SMD) were used to assess covariate balance between the two groups. An adequate balance was considered for SMD < 0.20 across covariates.

A sensitivity analysis considered a full sample of ICs, including those ICs with missing sociodemographic characteristics. Hot-deck imputation was used to impute missing data on race/ethnicity, education, and relationship to the care recipient. Sex and age were not imputed because all respondents reported their age group and sex.

Given the count nature of the outcome variables (number of days of poor mental or physical health), marginal structural negative binomial regression models estimated adjusted incidence rate ratios (IRRs) of reporting days of poor physical and mental health as a function of whether one provided care before (control) or after the COVID-19 NED (exposed). Another study used a negative binomial regression approach to estimate differences in mental health and somatic symptoms between short- and long-term caregivers, and non-caregivers [33]. All analyses were weighted according to BRFSS methodology and performed in SAS 9.4. Graphical representations were performed in RStudio 2022.12.0 + 353.

## Results

The study population consisted of 13,779 ICs who provided care over the previous 30 days and completed the BRFSS survey in 2019 or 2020. Out of those 13,779 ICs, 3,638 were excluded from the analysis because they provided care both before and during the COVID-19 pandemic or because it was unclear whether their care provision status overlapped between the period before and during COVID-19 pandemic. The final sample consisted of a total of 9,240 ICs, who provided complete data on their sociodemographic characteristics (age, sex, race-ethnicity, educational attainment, and relationship to the care recipient). A weighted 90.03% (N = 8,379) of the ICs in the sample provided care before the COVID-19 NED, and 9.97% (N = 861) provided care after the COVID-19 NED went into effect.

Table 1 presents the weighted demographic characteristics of the sample, both total and by the COVID-19 NED status. The 861 ICs who provided care during the COVID-19 pandemic were successfully matched with 1,722 controls (selected among those who provided care before the COVID-19 NED). An adequate covariate balance (i.e., all SMD < 0.20) was achieved using 2:1 propensity score matching (Supplementary Figs. 1–2), with a caliper of 0.11.

**Table 1:** Baseline characteristics of Informal Caregivers, (unweighted counts and weighted percentages) – Weighted complete case

		Period of care provision	
	Total Sample	Before COVID-19 NED	After COVID-19 NED
N	9,240 (100.00)	8,379 (90.03)	861 (9.97)
Age Category n (%)			
18–24	372 (7.59)	317 (7.14)	55 (11.71)
25–34	685 (11.64)	609 (11.11)	76 (16.46)
35–44	1,004 (15.13)	905 (15.25)	99 (14.09)
45–54	1,466 (17.98)	1,323 (17.92)	143 (18.50)
55–64	2,228 (22.92)	2,049 (23.64)	179 (16.40)
65+	3,485 (24.73)	3,176 (24.94)	309 (22.85)
Biological Sex n (%)			
Male	3,474 (42.28)	3,144 (42.40)	330 (41.11)
Female	5,766 (57.72)	5,235 (57.60)	531 (58.89)
Race Ethnicity n (%)			
Non-Hispanic, Black	744 (12.79)	656 (12.51)	88 (15.35)
Non-Hispanic, Multiracial	413 (1.70)	397 (1.64)	16 (2.21)
Non-Hispanic, Other races	702 (4.86)	681 (5.05)	21 (3.12)
Hispanic	743 (14.38)	709 (15.13)	34 (7.69)
Non-Hispanic, White	6,638 (66.27)	5,936 (65.68)	702 (71.62)
Education n (%)			
Did not graduate High School	543 (10.09)	479 (9.83)	64 (12.44)
Graduated High School	2,335 (28.02)	2,073 (27.87)	262 (29.37)
Attended college/Tech school	2,883 (34.80)	2,628 (34.90)	255 (33.96)
Graduated from College/Tech	3,479 (27.09)	3,199 (27.41)	280 (24.23)
Relation n (%)			
Sibling/Spouse/In-laws^(a)^	2,602 (24.96)	2,353 (25.13)	249 (23.39)
Other relatives or friends	2,465 (23.04)	2,182 (22.28)	283 (29.89)
Intergenerational^(b)^	4,173 (52.00)	3,844 (52.59)	329 (46.71)

Note: Unweighted count and weighted column percentages in parantheses from complete case data. ^(a)^ in-laws include brother- and sister-in-laws. ^(b)^ intergenerational includes father, mother, children, grandparents, parents-in-laws, and grandchildren

Table 2 presents a summary of weighted descriptive statistics across baseline characteristics for each outcome of interest in the matched data. Descriptively, the average number of days of poor physical health was observed to be higher among those who provided care before the COVID-19 NED (4.62 days) compared to those who provided care after the COVID-19 NED (3.41 days). However, the mean number of days of poor mental health observed was only slightly higher among those who provided care after the COVID-19 NED (5.68 days) compared to those who provided care before the COVID-19 NED (5.35 days).

**Table 2:** Weighted average number of days poor physical and mental health days by baseline characteristics (2:1 matched sample)

Informal Caregiver Characteristics	Number of days of poor physical health	Number of days of poor mental health
	Mean (Std. Error)	Mean (Std. Error)
**Caregiving before or after National Emergency Declaration (NED)**		
Provided care after COVID-19 NED	3.41 (0.43)	5.68 (0.52)
Provided care before COVID-19 NED	4.62 (0.35)	5.35 (0.43)
**Age Group**		
18–24	1.51 (0.44)	8.66 (1.24)
25–34	2.86 (0.49)	7.07 (1.20)
35–44	4.19 (0.79)	6.31 (0.84)
45–54	4.33 (0.77)	6.31 (0.96)
55–64	4.54 (0.66)	4.72 (0.63)
65+	5.04 (0.49)	3.19 (0.40)
**Biological Sex**		
Male	4.52 (0.51)	4.89 (0.56)
Female	3.82 (0.29)	5.90 (0.41)
**Race Ethnicity**		
Non-Hispanic Black	2.79 (0.55)	5.29 (1.15)
Non-Hispanic,Multiracial	2.49 (0.83)	8.16 (2.25)
Non-Hispanic, Other races	1.99 (0.85)	6.67 (2.61)
Hispanic	2.47 (0.89)	7.46 (2.23)
Non-Hispanic White	4.58 (0.32)	5.31 (0.31)
**Level of education**		
Did not graduate High School	4.04 (0.77)	5.96 (1.15)
Graduated High School	5.23 (0.65)	6.02 (0.61)
Attended college/Technical school	4.27 (0.44)	5.65 (0.53)
Graduated from College/Technical	2.68 (0.29)	4.56 (0.71)
**Relationship with care recipient**		
Sibling/Spouse/in-laws^(a)^	5.17 (0.61)	5.28 (0.59)
Other relatives or friends	3.83 (0.44)	5.50 (0.51)
Intergenerational ^(b)^	3.68 (0.38)	5.60 (0.55)

Note: ^(a)^ in-laws include brother- and sister-in-laws. ^(b)^ intergenerational includes father, mother, children, grandparents, parents-in-laws, and grandchildren

The reported mean number of days of poor physical and mental health differed across age groups: younger age ICs (i.e., < 25 years) reported a higher average number of days of poor mental health (8.66 days in this age group) compared to older ICs (3.19 days among those 65+). However, the average number of days of poor physical health was observed to be lower among those < 25 years (1.51 days) compared to those 65+ (5.04 days).

**Fig. 1:**
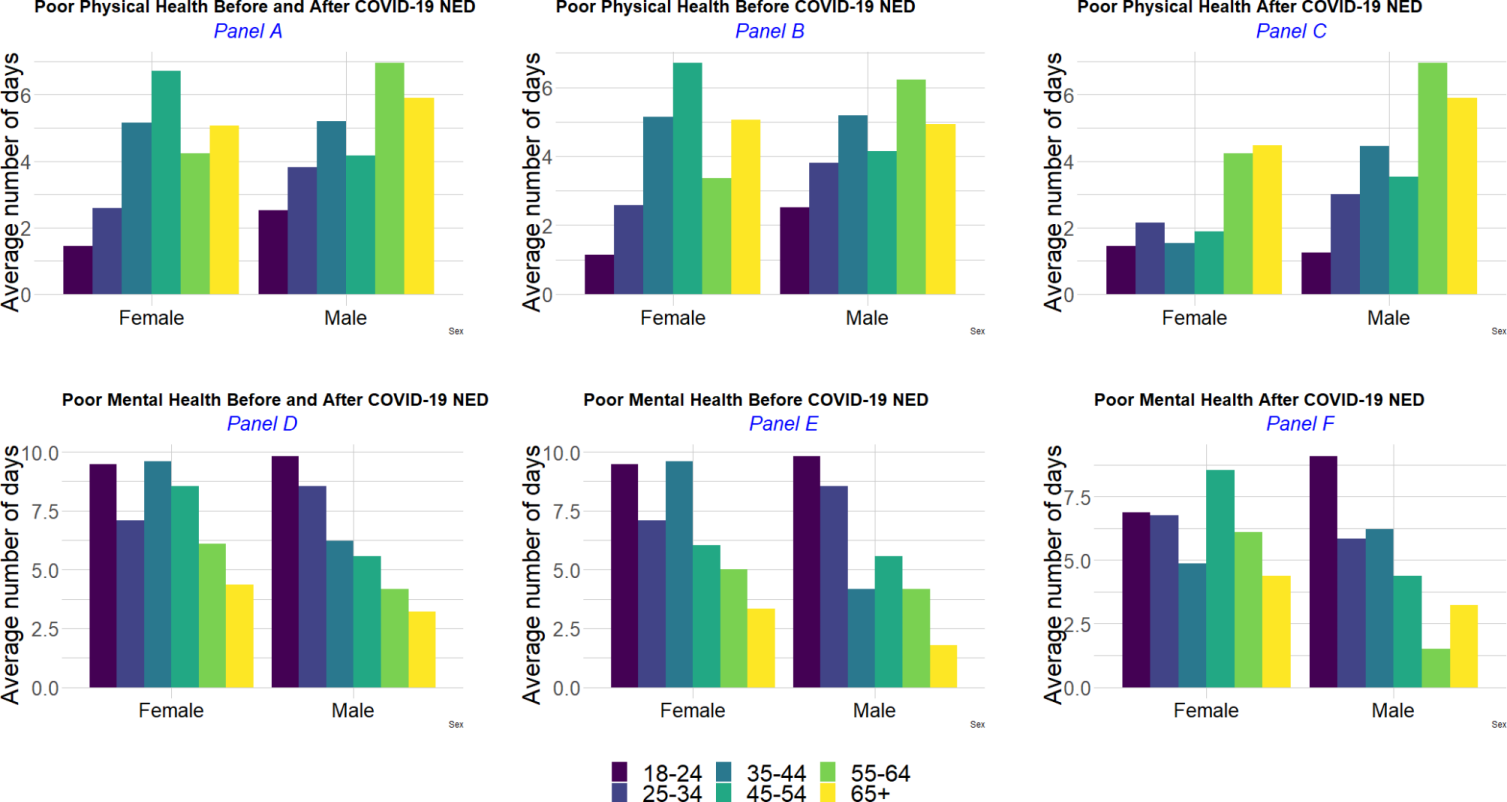
Weighted average number of days of poor physical and mental, health by sex and age

Male ICs were observed to have a slightly higher average number of days of poor physical health compared to female ICs (e.g., 4.52 versus 3.82 days); whereas female ICs reported a higher mean number of days of poor mental health compared to male ICs (e.g., 5.90 versus 4.89 days). Across all age groups, male ICs reported higher averages of poor physical health days compared to female ICs within the same age groups, except for female ICs aged 45–54 years, who reported a higher observed average number of days of poor physical health compared to male ICs of the same age group. However, female ICs consistently reported a higher observed average number of days of poor mental health compared to male ICs of similar age groups, and this trend held across all age groups, except for 25–34 years old group (Fig. 1). Compared to the period before the COVID-19 NED, the observed average number of days of poor physical health after the COVID-19 NED decreased in both sexes across most age groups; except for the youngest female ICs (18–24 years old); female ICs aged 55–64; and older male ICs (55 + years old). The observed average number of days of poor mental health decreased from its levels prior to the COVID-19 NED among younger female ICs (< 45 years old); whereas older female ICs (45 + years old) reported on average higher numbers of days of poor mental health, compared to before the COVID-19 NED. Among young male ICs (< 35 years old), the period following the COVID-19 NED saw a lower observed average number of days of poor mental health compared to its levels prior to the COVID-19 NED. However, among male ICs aged 35–44 and 65 years and older, the period following the COVID-19 NED recorded a higher observed average number of days of poor mental health compared to its levels prior to the COVID-19 NED (Fig. 1).

**Fig. 2:**
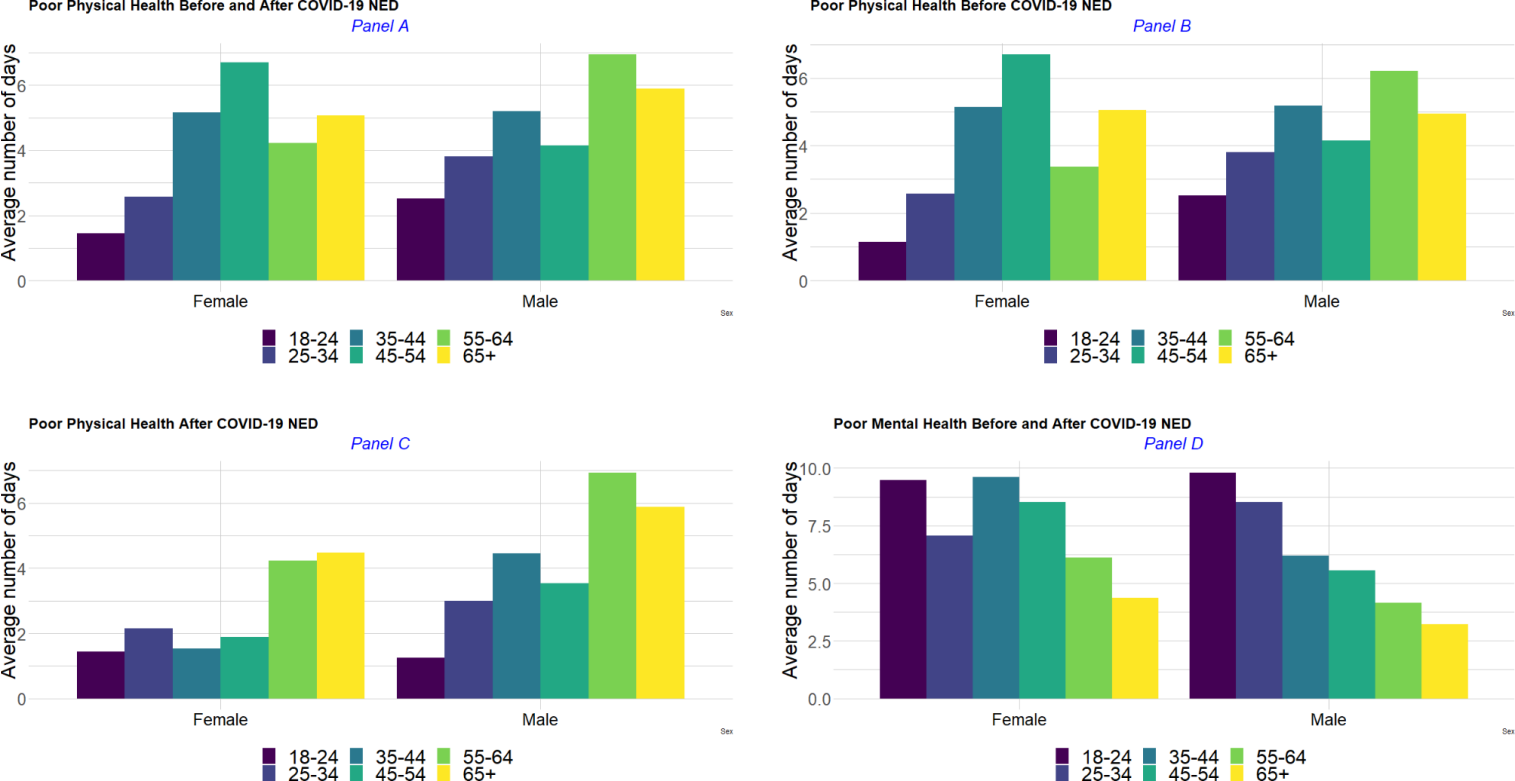
Weighted average number of days of poor physical and mental health by race-ethnicity, and by race-ethnicity across age groups

On average, non-Hispanic White ICs reported the highest number of days of poor physical health (4.58 days) and ICs who identified as non-Hispanic other races had the lowest observed average number of days of poor physical health (1.99 days) in the combined period. By contrast, non-Hispanic Black and White ICs had the lowest observed average number of days of poor mental health (5.29 and 5.31, respectively) compared to individuals with another racial/ethnic group identity. People who identified as non-Hispanic multiracial reported the highest number of days of poor mental health (8.16 days on average) (Fig. 2). Across race-ethnicity stratification, middle-age [35–44] ICs self-identified as non-Hispanic other races and older (55 + years of age), non-Hispanic multiracial ICs had the lowest observed average numbers of days of poor physical and physical health. Older non-Hispanic other races ICs (65+) had the highest observed average number of days of poor physical health (on average 9.7 days of poor physical health), compared to other race-ethnicity groups. The highest average number of days of poor mental health was reported among younger ICs (18–24 years old group) self-identifying as non-Hispanic other races, as well as Hispanic ICs aged 25–34; whereas the lowest average number of days of poor mental health was recorded among older Hispanic (65 + years) and non-Hispanic multiracial ICs (55 + years of age).

**Fig. 3:**
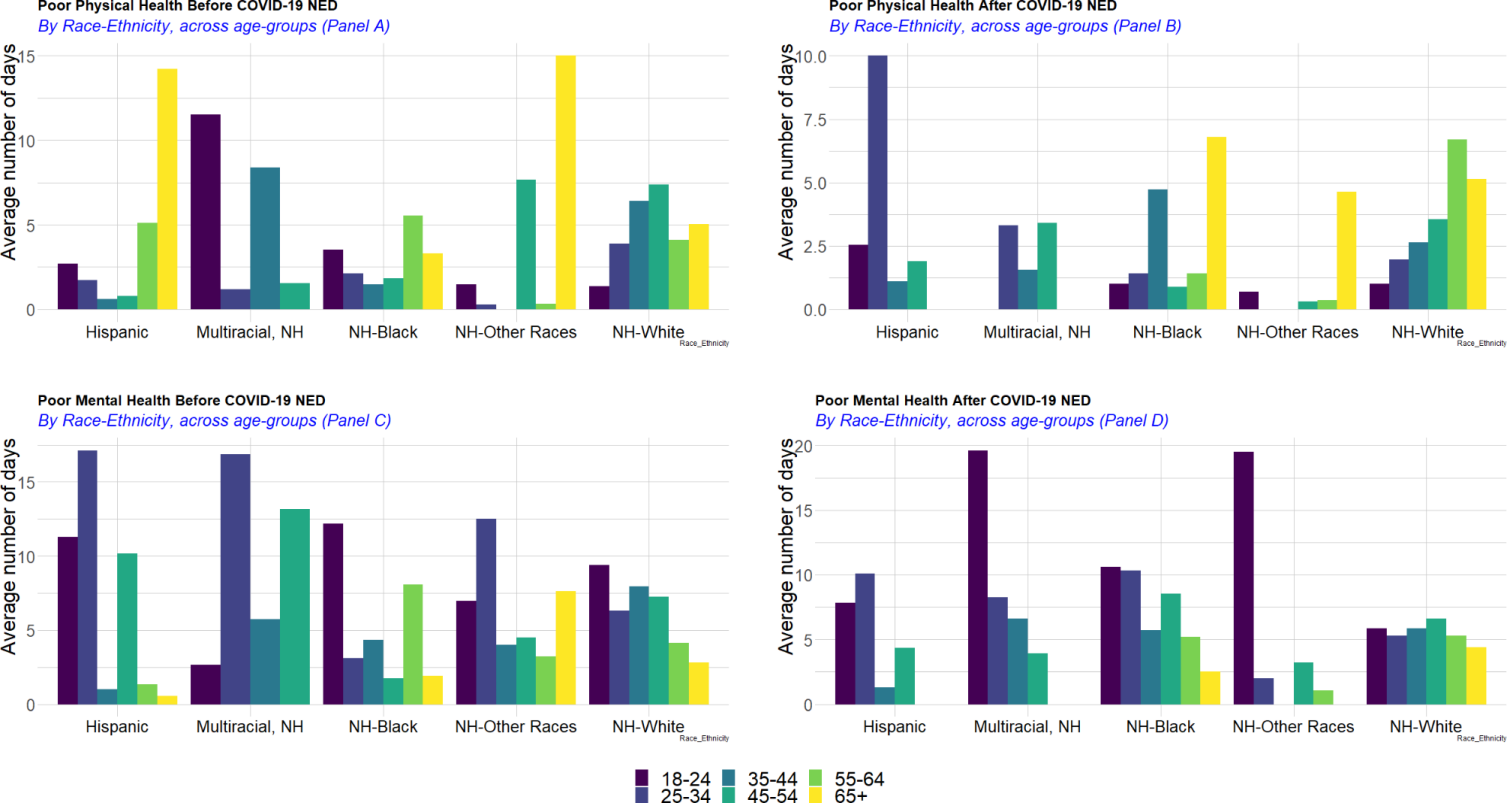
Weighted average number of days of poor physical and mental health, before and after the COVID-19 NED

Prior to the COVID-19 NED, older ICs (65 + years of age) of Hispanic origins and non-Hispanic other races had the highest observed average number of days of poor physical health (on average more than 13 days of poor physical health), however after the COVID-19 NED older (55 + years) Hispanic ICs and middle age [25–44] ICs of non-Hispanic other race origins had the lowest average number of days of poor physical health (Fig. 3, panels A and B), though the numbers of respondents are smaller for some of these subpopulations. Hispanic ICs aged 25–34 experienced the biggest increase in the average number of days of poor physical health (from 1.71 days to 10.00 days); also, non-Hispanic Black ICs aged 35–44 and 65 + years and older non-Hispanic White (55 + years of age) reported higher numbers of days of poor physical health after the COVID-19 NED compared to the period before the COVID-19 NED.

In terms of mental health (Fig. 3, panels C and D), Hispanic and multiracial non-Hispanic ICs aged 25–34 reported the highest average number of days of poor mental health before the COVID-19 NED. After the COVID-19 NED, young (< 25 years) non-Hispanic multiracial and non-Hispanic ICs of other races experienced the highest average number of days of poor mental health. Across the other racial-ethnic groups, the average number of days of poor mental health increased among non-Hispanic Black (e.g., 25–54; and 65 + years of age) and older non-Hispanic White (55 + years of age).

### Incidence rate ratios of reported days of poor physical health

Table 3 includes adjusted incidence rate ratios and corresponding p-values from the weighted negative binomial regressions using the 2:1 matched sample. The adjusted incidence rate of poor physical health days was 26% lower (p = 0.001) among ICs who provided care after versus before the COVID-19 NED. The incidence rate of reporting days of poor physical health was 69% lower (p < 0.001) among ICs aged 18–24 compared to those aged 65+. The incidence rate for reporting days of poor physical health was 44% lower (p = 0.001) among ICs aged 25–34 compared to those aged 65+. The incidence rates of reporting days of poor physical health were higher for ICs with lower educational attainment (e.g., 2.03; and 1.68 times higher, respectively, for those with no high school diploma, those with high school diploma only, and those who attended college or technical school without obtaining a degree, respectively) compared to those who graduated from college or technical school). The incidence rate of reporting days of poor physical health was 30% lower (p = 0.005) among non-Hispanic Black ICs compared to non-Hispanic White ICs. The incidence rate of reporting days of poor physical health was 46% (p = 0.042) among those identifying as non-Hispanic of other races compared to non-Hispanic White. The relationship with the care recipient was non-significant (p ≥ 0.844).

### Incidence rate ratios of reported days of poor mental health

The adjusted incidence rate of reported days of poor mental health was not statistically different (p = 0.730) between informal caregiving before versus after the COVID-19 NED, nor across racial/ethnic identities (p > 0.591) when compared to non-Hispanic White individuals. The incidence rate of reported days of poor mental health stood higher at younger ages compared to older ages. For example, ICs aged 18–24 had 3.32 times higher incidence rates than those aged 65 years and older (p < 0.001). Similar results were found across age groups, with smaller differences as age gaps narrowed.

The incidence rate of reported days of poor mental health was 29% lower (p < 0.001) among male ICs compared to female ICs. Those with generally lower education had higher incidence rates of reporting days of poor mental health compared to graduates from college or technical school (p ≤ 0.012), although the comparison with those who did not graduate from high school was non-significant (p = 0.095). Furthermore, the incidence rate of poor mental health days was 37% higher for IC for a sibling, spouse, or brothers- and sisters-in-law (p = 0.004) compared to that of intergenerational relatives (including father, mother, child, grandparents, parents-in-law, and grandchild); whereas no significant difference was found for IC to other relatives or friends.

Supplementary Figs. 3–4 portray the residual plots for both models.

**Table 3:** Incidence rate ratios (IRRs) from the weighted negative binomial regressions using the 2:1 matched sample

Informal Caregiver Characteristic	Days of poor physical health		Days of poor mental health	
	IRR	p-value	IRR	p-value
**Caregiving period**				
Provided care after COVID-19 NED	0.74	0.001	1.03	0.730
Provided care before COVID-19 NED	Ref.		Ref.	
**Age Group**				
18–24	0.31	< 0.001	3.32	< 0.001
25–34	0.56	0.001	2.75	< 0.001
35–44	0.82	0.169	2.41	< 0.001
45–54	0.94	0.657	2.30	< 0.001
55–64	0.87	0.294	1.54	< 0.001
65+	Ref		Ref.	
**Biological Sex**				
Male	1.19	0.063	0.71	< 0.001
Female	Ref.		Ref.	
**Race Ethnicity**				
Non-Hispanic, Black	0.70	0.005	1.01	0.932
Non-Hispanic, Multiracial	0.80	0.585	1.02	0.966
Non-Hispanic, Other races	0.54	0.042	1.05	0.845
Hispanic	1.02	0.929	0.89	0.591
Non-Hispanic, White	Ref.		Ref.	
**Level of education**				
Did not graduate High School	2.03	< 0.001	1.32	0.095
Graduated High School	2.03	< 0.001	1.41	0.012
Attended college/Technical school	1.68	< 0.001	1.30	0.002
Graduated from College/Tech school	Ref		Ref.	
**Relationship with care recipient**				
Sibling/spouse/or in-laws^(a)^	1.02	0.899	1.37	0.004
Other relatives or friends	0.98	0.844	1.11	0.287
Intergenerational^(b)^	Ref.		Ref.	

Note: ^(a)^ in-laws include brother- and sister-in-laws. ^(b)^ intergenerational includes father, mother, children, grandparents, parents-in-laws, and grandchildren

### Sensitivity analysis

Results from the hot-deck imputed data were comparable to those in the complete case analysis. There were 448 missing observations imputed from the original sample of ICs, who provided care before or during the COVID-19 pandemic but had missing sociodemographic characteristics. Overall, a weighted 10.72% of the ICs provided care after the COVID-19 NED upon imputation, compared to 9.97% in the main analysis (Supplementary Table 1). Covariate balance was achieved using propensity score matching, with all standardized mean differences below 0.20 (Supplementary Fig. 5).

In the imputed, 2:1 matched sample, 1,003 ICs who provided care after the COVID-19 NED were successfully matched to 2,006 controls (ICs who provided care before the COVID-19 NED). Like in the primary analysis, the average number of days of poor physical health was observed to be higher (5.16 days) among ICs who provided care before the COVID-19 NED compared to those who provided care after the COVID-19 NED (3.72 days) (Supplementary Table 2). Days of poor mental health were higher (6.28 days) among those who provided care after the COVID-19 NED compared to those who provided care before the COVID-19 NED (5.40 days). At younger age ranges, ICs reported fewer days of poor physical health, but more days of poor mental health, whereas at older ages this was reversed. Likewise, ICs with low educational attainment reported on average a higher number of days of poor physical and mental health.

Results from the negative binomial regression models (Supplementary Table 3) showed similar results to that of the primary analysis – i.e., that although informal caregiving during the COVID-19 pandemic was associated with decreased incidence rates of days of poor physical health (p < 0.001), there was no significant difference in the incidence of poor mental health days (p = 0.075) among ICs before versus after the COVID-19 NED. In comparison, at younger ages (all age groups less than 65 years old; all p < 0.001), the incidence rate of reported poor mental health days was higher than for those aged 65+. Lower educational attainment was similarly associated with a higher incidence rate of reported poor mental and physical health days (all p-values < 0.001 across all education levels compared to those with a college or technical school degree).

## Discussion

Informal caregiving during the COVID-19 pandemic was associated with a lower incidence of reported days of poor physical health when compared to the pre-pandemic period. The reported number of days of poor mental health was higher among ICs who provided care during the COVID-19 pandemic and those who provided care before the COVID-19 NED. However, results from our final model suggest that the incidence rate for days of poor mental health was not statistically different between ICs who provided care before COVID-19 NED and those who provided care after COVID-19 NED. Though COVID-19 NED and its subsequent string of restrictions created societal stress (which we conceptualized as the contextual stimulus), analyses in this study suggest that it did not negatively impact the physical and mental health of ICs in the sense of the caregiver stress model. Although this finding does not align with many other studies that have reported deteriorated mental health among ICs during COVID-19 [34–36], our finding contends that the impact of a contextual stimulus (e.g., COVID-19 NED) on the health of the caregivers cannot be captured by focusing merely on on aspects of the stress model (e.g., the contextual stimulus itself, either the objective or subjective burden) [34]. Instead, we found that different groups of ICs were impacted differently. In comparison to those who provided care before the COVID-19 NED, ICs who provided care during the COVID-19 pandemic reported a statistically significant lower number of days of poor physical health. This difference may reflect the flexible work schedule afforded to some individuals during lockdowns and work-from home policies enacted by several organizations across the United States that resulted in anticipated substantially lower exposure to non-COVID transmissible diseases when compared to pre-NED periods [37–40]. However, there were significant differences in the incidence of reported days of poor physical health across age groups and education. Younger (18–24 and 25–34) ICs reported fewer number of days of poor physical health compared to older aged (65+) ICs. Among all age groups, male ICs reported a higher number of days of poor physical health on average compared to female ICs, except for male ICs aged 45–54 who reported fewer poor physical health days compared to women. That female ICs experienced a higher number of days of poor physical health compared to male ICs aged 45–54 may reflect a menopause transition. For instance, another study assessed changes in caregiver burden and intensity due to COVID-19 among informal caregivers aged 50 + and found significant differences in the increased caregiver burden and intensity due to COVID-19 between male and female informal caregivers [41]. Another U.S. Study of Women’s Health Across the Nation SWAN reported that menopause transition is associated with mental health concerns (e.g., vasomotor symptoms, sleep complaints, urogenital and sexual health complaints) and physiological systems and functions (e.g., cardiovascular and cardiometabolic health, bone health, and physical function performance) [42].

Lower educational attainment was associated with higher incidence rates of days of poor physical health, which stresses the role education, and subsequently income, can play in promoting healthy people [43]. In addition, compared to non-Hispanic White ICs, non-Hispanic Black ICs and non-Hispanic ICs of another race had lower incidence rates of days of poor physical health. This difference could reflect age differences across race-ethnicity among ICs, because the proportion of non-Hispanic White ICs increased with increasing age groups, suggesting that non-Hispanic White ICs were, on average, older than other race-ethnicity groups. More research is needed to parse out variations in health outcomes among ICs by race and ethnicity and other social determinants of health.

Male ICs had a lower incidence rate of reported days of poor mental health compared to female ICs. These findings align with multiple other studies, which suggest that emotional strain may be greater among women than men ICs, because women may feel overloaded with other household tasks in addition to the informal caregiving activities [13, 16, 44, 45].

ICs’ mental and physical health differed by age and sex. As one might expect, physical health issues increase with age, and therefore younger ICs reported lower incidence rates of poor physical health compared to older ICs; yet they reported higher incidence rates of poor mental health days compared to older caregivers. This finding may point to ineffective coping skills at younger ages [46], greater risks of becoming unemployed and associated financial strains during the lockdown [47], and restrictions imposed on their more active social lifestyle, which included lack of access to gym facilities, eating healthy, missing doctor appointments, and other restrictions [48].

We also found that educational level of ICs was associated with mental health outcomes: participants who graduated from college or technical schools experienced lower incidence rates of poor mental health days. This could point to a potential lack of access to resources and access to the types of social support (informational, emotional, appraisal, instrumental, etc.) afforded by the networks of those with lower educational attainment. Educational attainment has been documented in a previous study as a confounding factor for both physical and mental health during the COVID-19 pandemic [49].

### Limitations

Limitations in this study relate to the ability to accurately categorize those who provided care before and after the COVID-19 NED. Among those who completed the survey after March 13th, 2020, some had been providing care to their loved ones prior to the NED. However, the variable that defined the length of time during which the informal caregiver had been providing care made it challenging to approximate when exactly they started their informal caregiving. Nevertheless, the cross-sectional nature of the survey allowed for investigation on the current experience of ICs at the time of survey. Another limitation is that types of care provided may not be homogeneous in the two groups, since informal care provision could have been provided to COVID-19-recovering patients, who would not appear in the control group. The unknown surrounding variations in types of caregiving for patients with COVID-19 during the early pandemic stages could be an unaccounted-for confounder. The length of informal caregiving experience is also unknown, and the study did not control for the numbers of hours spent on caregiving, which would have captured the intensity of care provision. ICs with more experience previously providing care may have developed higher resilience than those who more recently became an IC. Another limitation to this study is related to the aggregation of the racial-ethnic groups under other. While this aggregation was done for analytical purposes to overcome concerns related to small sample size, we recognize that cultural differences between ethnic groups may be different. We also recognize that racial-ethnic groups as captured in the data can contain high levels of heterogeneity. For instance, some racial-ethnic groups may find it customary to help their loved ones who need care through a family-wide approach to IC, while for other racial-ethnic groups, this may be less common. While a study strength involves the analysis of national data, a noteworthy challenge surrounding this is that COVID-19 restrictions varied widely across states and jurisdictions, especially in the latter part of 2020; hence, it was not feasible to control for such variability in this national study. Finally, while we controlled for some important sociodemographic covariates, there is a wide range of additional sociodemographic factors potentially associated with physical and mental health outcomes, including the specific characteristics and health risks/comorbidities of ICs themselves.

## Conclusions

The mental and physical health burden associated with informal caregiving may be heightened during a global pandemic which is a period of great uncertainty. We found that the incidence rate for days of poor physical health was lower among ICs who provided care during the COVID-19 pandemic, however their incidence rate for days of poor mental health was not significantly different from those who provided care prior to the COVID-19 NED. Age and educational attainment of ICs were significant for both outcomes. ICs with lower education levels tended to report higher poor mental and physical health days compared to graduates from college or technical school. Days of poor physical and mental health were lower among ICs aged 18–24 compared to those aged 65+. Future studies should compare the physical and mental health outcomes among ICs before and during the COVID-19 pandemic, at the county, district, and state level as additional variability may be hidden in aggregate analyses. Relatedly, policy makers should consider expanding access to resources through institutional mechanisms for ICs in most need, who may be likely to incur a higher physical and mental health burden during national emergencies. The question relative to the duration of care provision by IC would allow a better assessment of the impact of duration on health, if answers were provided on a discrete scale rather than the current categories.

### Electronic supplementary material

Below is the link to the electronic supplementary material.

Supplementary Material 1

## Data Availability

Datasets analyzed during the current study are available in the Centers for Disease Control and Prevention Behavioral Risk Factor Surveillance Survey repository: https://www.cdc.gov/brfss/annual_data/annual_2020.html.
