# Supplementary material for: Physical and mental health of informal caregivers before and during the COVID-19 pandemic in the United States
Source: BMC Public Health. 2023 Nov 27;23:2349. doi: 10.1186/s12889-023-17164-8 (PMC10683238; doi:10.1186/s12889-023-17164-8)
Supplement: Supplementary file 1 — Supplementary Material 1 [file 12889_2023_17164_MOESM1_ESM.docx]

**Supplementary file**


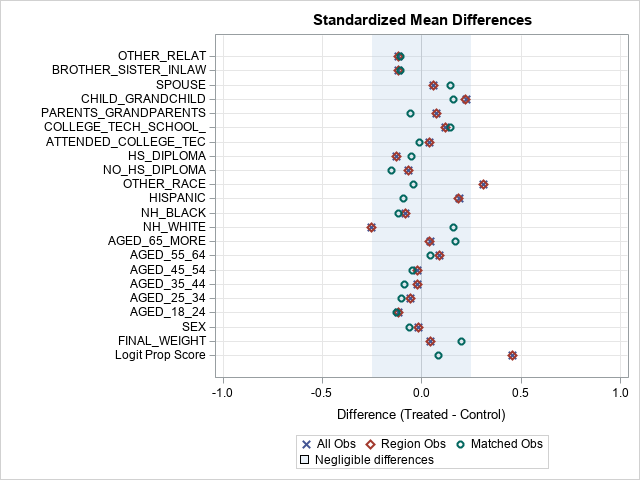


**Supplementary Figure 1**: Standardized mean difference by covariate for complete case data after matching


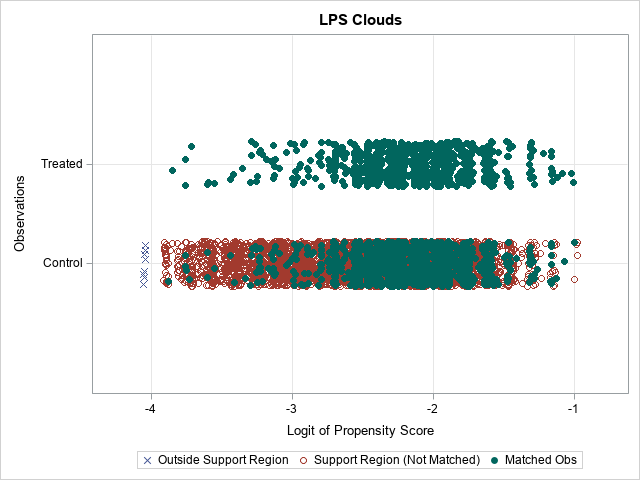


**Supplementary Figure 2**: Logit propensity score (LPS) cloud plot by group for complete case data after matching


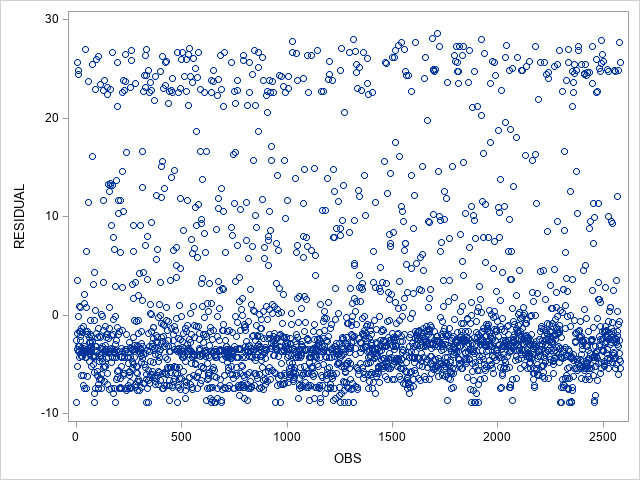


**Supplementary Figure 3**: Residual plot for the model estimating number of days of poor physical health (matched data)


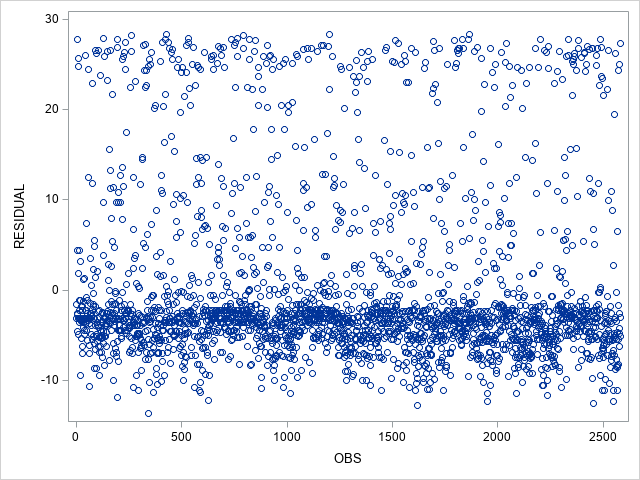


**Supplementary Figure 4**: Residual plot for the model estimating number of days of poor mental health (matched data)


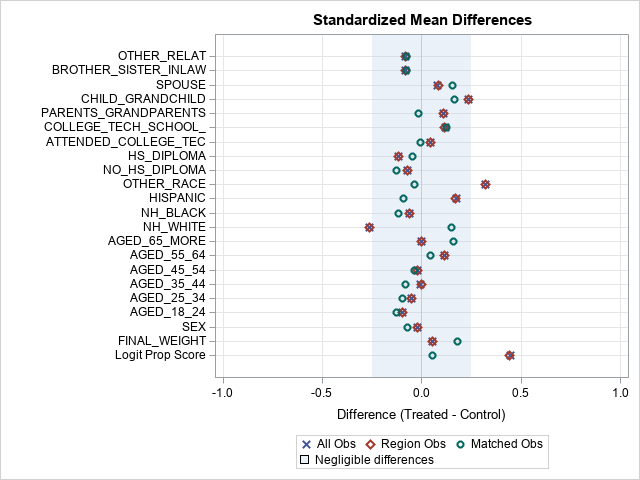


**Supplementary Figure 5**: Standardized mean difference by covariate for matched data after imputation (n=2,006 cases matched)

**Supplementary Table 1: Weighted baseline characteristics of informal caregivers (unweighted counts and weighted percentages) from imputed data**

|  |  | Period of care provision | |
| --- | --- | --- | --- |
|  | Total Sample | Before COVID-19 NED | After COVID-19 NED |
| N | **10,141 (100.0)** | **9,138 (89.28)** | **1,003 (10.72)** |
| Age Category (%) |  |  |  |
| 18-24 | 423 (7.6) | 362 (7.17) | 61 (11.03) |
| 25-34 | 774 (12.2) | 684 (11.63) | 90 (16.68) |
| 35-44 | 1,110 (15.2) | 990 (15.33) | 110 (14.38) |
| 45-54 | 1,630 (18.1) | 1,461 (18.02) | 169 (19.02) |
| 55-64 | 2,430 (22.4) | 2,232 (23.25) | 198 (14.92) |
| 65+ | 3,784 (24.5) | 3,409 (24.60) | 375 (23.98) |
| Sex (%) |  |  |  |
| Male | 3,835 (42.6) | 3,446 (42.9) | 389 (40.7) |
| Female | 6,306 (57.4) | 5,692 (57.1) | 614 (59.3) |
| Race Ethnicity (%) | | |  |
| Non-Hispanic, Black | 854 (13.0) | 753 (12.9) | 101 (14.2) |
| Non-Hispanic, Multiple Race | 458 (1.8) | 440 (1.72) | 18 (2.2) |
| Non-Hispanic, Other races | 816 (14.5) | 762 (5.3) | 23 (3.2) |
| Hispanic | 785 (5.0) | 774 (15.1) | 42 (9.5) |
| Non-Hispanic, White | 7,228 (65.7) | 6,409 (65.0) | 819 (70.9) |
| Education (%) |  |  |  |
| Did not graduate High School | 609 (10.2) | 533 (9.8) | 76 (12.9) |
| Graduated High School | 2,584 (27.7) | 2,280 (27.6) | 304 (28.5) |
| Attended college/Tech school | 3,163 (35.3) | 2,866 (35.5) | 297 (33.9) |
| Graduated from College/Tech | 3,785 (26.9) | 3,459 (27.1) | 326 (24.7) |
| Relation (%) |  |  |  |
| Sibling/Spouse/in-laws^(a)^ | 2,822 (24.6) | 2,538 (24.8) | 284 (22.3) |
| Other relatives | 2,742 (23.8) | 2,410 (23.0) | 332 (29.4) |
| Intergenerational^(b)^ | 4,577 (51.8) | 4,190 (52.2) | 387 (48.3) |

Note: ^(a)^ in-laws include brother- and sister-in-laws. ^(b)^ include father, mother, children, grandparents, parents-in-laws, and grandchildren

**Supplementary Table 2: Weighted average number of poor physical and mental health days by baseline characteristics (2:1 matched sample and imputed data)**

| **Characteristics** | **Number of poor physical days** | **Number of poor mental health days** |
| --- | --- | --- |
|  | **Mean (Std. Error)** | **Mean (Std. Error)** |
| **Caregiving before or after declared national emergency** |  |  |
| Provided care before COVID-19 | 5.16 (0.37) | 5.40 (0.39) |
| Provided care during COVID-19 | 3.72 (0.48) | 6.28 (0.58) |
| **Age Group** |  |  |
| 18-24 | 2.62 (0.71) | 9.15 (1.20) |
| 25-34 | 2.88 (0.46) | 7.31 (1.12) |
| 35-44 | 5.47 (1.00) | 7.18 (1.19) |
| 45-54 | 5.12 (0.86) | 5.72 (0.73) |
| 55-64 | 5.18 (0.71) | 5.61 (0.78) |
| 65+ | 4.75 (0.42) | 3.26 (0.35) |
| **Sex** |  |  |
| Male | 4.87 (0.48) | 5.01 (0.59) |
| Female | 4.36 (0.37) | 6.25 (0.40) |
| **Race Ethnicity** |  |  |
| Non-Hispanic, Black | 3.46 (0.73) | 5.30 (1.01) |
| Multiracial, Non-Hispanic | 3.11 (0.93) | 9.88 (2.63) |
| Non-Hispanic, Other races | 3.49 (1.09) | 6.71 (2.31) |
| Hispanic | 3.78 (1.36) | 8.94 (2.48) |
| Non-Hispanic, White | 4.89 (0.34) | 5.51 (0.33) |
| **Level of education** |  |  |
| Did not graduate High School | 4.34 (0.88) | 9.04 (1.75) |
| Graduated High School | 6.06 (0.65) | 6.28 (0.61) |
| Attended college/Technical school | 4.76 (0.54) | 5.95 (0.51) |
| Graduated from College/Technical | 2.81 (0.28) | 3.80 (0.51) |
| **Relationship with care recipient** |  |  |
| Sibling/Spouse/in-laws^(a)^ | 4.81 (0.53) | 6.15 (0.57) |
| Other relatives or friends | 4.11 (0.40) | 5.51 (0.59) |
| Intergenerational ^(b)^ | 4.67 (0.49) | 5.30 (0.46) |

Note: ^(a)^ in-laws include brother- and sister-in-laws. ^(b)^ include father, mother, children, grandparents, parents-in-laws, and grandchildren

**Supplementary Table 3: Incidence rate ratios (IRR) from the weighted negative binomial using the 2:1 matched sample and imputed data**

| **Characteristics** | **IRR** | **p-value** | **IRR** | **p-value** |
| --- | --- | --- | --- | --- |
| **Caregiving period** |  |  |  |  |
| Provided care after COVID-19 NED | 0.68 | <0.001 | 1.15 | 0.075 |
| Provided care before COVID-19 NED | Ref |  | Ref. |  |
| **Age Group** |  |  |  |  |
| 18-24 | 0.46 | <0.001 | 2.95 | <0.001 |
| 25-34 | 0.55 | <0.001 | 2.52 | <0.001 |
| 35-44 | 1.01 | 0.907 | 2.33 | <0.001 |
| 45-54 | 1.06 | 0.673 | 1.88 | <0.001 |
| 55-64 | 0.99 | 0.967 | 1.60 | <0.001 |
| 65+ | Ref |  | Ref. |  |
| **Sex** |  |  |  |  |
| Male | 1.11 | 0.223 | 0.66 | <0.001 |
| Female | Ref. |  | Ref. |  |
| **Race Ethnicity** |  |  |  |  |
| Non-Hispanic, Black | 0.72 | 0.011 | 0.93 | 0.513 |
| Non-Hispanic, Multiracial | 0.94 | 0.875 | 1.32 | 0.371 |
| Non-Hispanic, Other races | 0.84 | 0.510 | 1.02 | 0.920 |
| Hispanic | 1.08 | 0.698 | 1.14 | 458 |
| Non-Hispanic, White | Ref. |  | Ref |  |
| **Level of education** |  |  |  |  |
| Did not graduate High School | 1.88 | <0.001 | 1.13 | <0.001 |
| Graduated High School | 2.28 | <0.001 | 1.66 | <0.001 |
| Attended college/Technical school | 1.74 | <0.001 | 1.58 | <0.001 |
| Graduated from college/Tech school | Ref. |  | Ref. |  |
| **Relationship with care recipient** |  |  |  |  |
| Sibling/Spouse/in-laws^(a)^ | 1.94 | 0.583 | 1.22 | 0.048 |
| Other relatives or friends | 1.01 | 0.911 | 1.03 | 0.746 |
| Intergenerational ^(b)^ | Ref. |  | Ref. |  |

Note: ^(a)^ in-laws include brother- and sister-in-laws. ^(b)^ include father, mother, children, grandparents, parents-in-laws, and grandchildren
